# Physiological Hypoxia and Pharmacological HIF Stabilization Induce Distinct but Overlapping Responses in Retinal Pigment Epithelial Cells

**DOI:** 10.3390/ph19091355

**Published:** 2026-08-27

**Authors:** Tamás Gáll, Dávid Pethő, Szilárd Póliska, József Balla, György Balla

**Affiliations:** 1 Division of Nephrology, Department of Internal Medicine, Faculty of Medicine, University of Debrecen, H-4032 Debrecen, Hungary; 2 HUN-REN–UD Vascular Biology and Myocardium Pathophysiology Research Group, Hungarian Academy of Sciences, University of Debrecen, H-4032 Debrecen, Hungary; 3 Genomic Medicine and Bioinformatic Core Facility, Department of Biochemistry and Molecular Biology, Faculty of Medicine, University of Debrecen, H-4032 Debrecen, Hungary; poliska@med.unideb.hu; 4 Department of Pediatrics, Faculty of Medicine, University of Debrecen, H-4032 Debrecen, Hungary

**Keywords:** retinal pigment epithelial cells (RPE), hypoxia, HIF-prolyl hydroxylase inhibitors (HIF-PHIs), RNA sequencing, VEGFA, metabolic reprogramming

## Abstract

**Background/Objectives:** Hypoxia is a major contributor to the development of retinal diseases by promoting metabolic adaptation and pathological angiogenesis through hypoxia-inducible factor (HIF) signaling. HIF-prolyl hydroxylase inhibitors (HIF-PHIs), which stabilize HIF under normoxic conditions, are widely used to treat anemia associated with chronic kidney disease. However, it remains unclear whether pharmacological HIF stabilization fully reproduces the cellular responses induced by physiological hypoxia. **Methods:** In this study, ARPE-19 cells were exposed to either physiological hypoxia or HIF-PHI treatment and analyzed using RNA sequencing, pathway enrichment, and pharmacological inhibition of PI3K/Akt, mTOR, and HIF-related signaling pathways. **Results:** Both treatments elicited a common transcriptional response marked by the induction of glycolytic and hypoxia-responsive genes together with increased vascular endothelial growth factor a VEGFA expression. Despite these similarities, only a small number of genes differed between the two conditions, suggesting that physiological hypoxia activates additional regulatory mechanisms beyond HIF stabilization alone. Rapamycin inhibited VEGFA production only under physiological hypoxia, further supporting mechanistic differences between the two models. Functionally, conditioned media from hypoxic ARPE-19 cells promoted endothelial tube formation, which was significantly reduced by pathway inhibition. **Conclusions:** Collectively, these findings show that HIF-PHIs reproduce many, but not all, aspects of the physiological hypoxic response in RPE cells.

## 1. Introduction

The retinal pigment epithelium (RPE) is a specialized monolayer of polarized cells that maintains the outer blood–retinal barrier and supports metabolic homeostasis of photoreceptors through regulated nutrient transport [1]. It also contributes to the visual cycle and continuously phagocytoses photoreceptor outer segments, enabling renewal of the photoreceptor layer [1]. In addition, the RPE is an important source of pro-angiogenic factors and plays a central role in retinal and choroidal neovascularization [2]. Dysregulation of RPE function is therefore implicated in several retinal diseases, including diabetic retinopathy (DR) [3], age-related macular degeneration (AMD) [4], proliferative vitreoretinopathy [5], retinitis pigmentosa [6], and Stargardt disease [7], underscoring the need to understand the molecular mechanisms governing RPE homeostasis under physiological and disease conditions.

The retina is particularly sensitive to hypoxia because of its exceptionally high metabolic and oxygen (O_2_) demands, driven primarily by the continuous energy requirements of photoreceptors to support the conversion of light stimuli into neuronal signals [8,9]. Maintenance of retinal function requires a continuous O_2_ supply and efficient mechanisms for sensing and adapting to hypoxia [10]. Because the retina has a limited O_2_ reserve, hypoxia can rapidly disrupt neuroretinal function and contribute to degeneration and vision loss. Retinal O_2_-sensing pathways coordinate adaptive responses, including changes in blood flow, metabolic adjustments, and angiogenesis, to restore the balance between O_2_ supply and demand [10]. Accordingly, retinal function depends on a continuous and adequate supply of O_2_ and nutrients through the retinal and choroidal circulations [9]. Impaired perfusion resulting from altered hemodynamics or vascular dysfunction can disrupt this supply, leading to retinal ischemia and hypoxia [9].

Sustained hypoxia is recognized as a key pathological driver in retinal diseases, including AMD and DR [11,12]. Under hypoxia, cells activate adaptive stress responses largely controlled by hypoxia-inducible factors (HIFs) [11]. Defining how these pathways are modulated is important for understanding retinal adaptation and for identifying potential therapeutic targets.

During hypoxia, stabilization of hypoxia-inducible factor-1α (HIF-1α) and its translocation to the nucleus initiate a transcriptional program that supports metabolic adaptation and cell survival [11,13]. A major downstream effector is vascular endothelial growth factor A (VEGFA), which regulates angiogenesis [12]. While basal VEGFA activity is necessary for maintaining choroidal vascular integrity, excessive VEGFA expression under hypoxic conditions drives pathological neovascularization [12,14]. In parallel, hypoxic RPE cells shift their metabolism away from oxidative phosphorylation toward glycolysis [13].

Hypoxia-inducible factor prolyl hydroxylase inhibitors (HIF-PHIs) are orally administered agents used to treat anemia in chronic kidney disease (CKD). They act by preventing HIF degradation under normoxic conditions, thereby enhancing erythropoietin production and regulating iron metabolism [15]. Several HIF-PHIs, including Roxadustat and Dimethyloxalylglycine, have been reported to reach retinal tissue [16]. Experimental evidence further suggests that Roxadustat can induce retinal metabolic dysfunction and neurodegeneration, raising concerns about potential long-term retinal effects in CKD patients receiving HIF-PHI therapy [17].

Although HIF-PHIs mimic an important component of the cellular response to hypoxia by stabilizing HIF, physiological hypoxia also activates additional O_2_-sensitive pathways that are independent of HIF signaling [18]. Therefore, pharmacological HIF stabilization may not fully recapitulate the molecular and functional responses of RPE cells to hypoxia.

ARPE-19 cells are a widely used an in vitro model of the human retinal pigment epithelium, owing to their accessibility, ease of culture, and expression of several RPE-associated markers. Although they do not fully recapitulate the phenotype and functional characteristics of primary or mature RPE cells, ARPE-19 cells remain a valuable model for preliminary mechanistic studies and hypothesis generation. Accordingly, this study compared physiological hypoxia with HIF-PHI-induced HIF stabilization in ARPE-19 cells to evaluate the extent to which pharmacological HIF activation reproduces hypoxia-induced transcriptional, metabolic, and angiogenic responses. RNA sequencing was used to profile and compare pathway-level changes under both conditions, with emphasis on HIF signaling, metabolic reprogramming, and angiogenic regulation.

The study further builds on this approach by directly examining overlapping and distinct regulatory mechanisms between physiological hypoxia and HIF-PHI treatment in RPE cells.

## 2. Results

### 2.1. Optimization and Validation of Hypoxic Culture Conditions for ARPE-19 Cells

**Figure 1:**
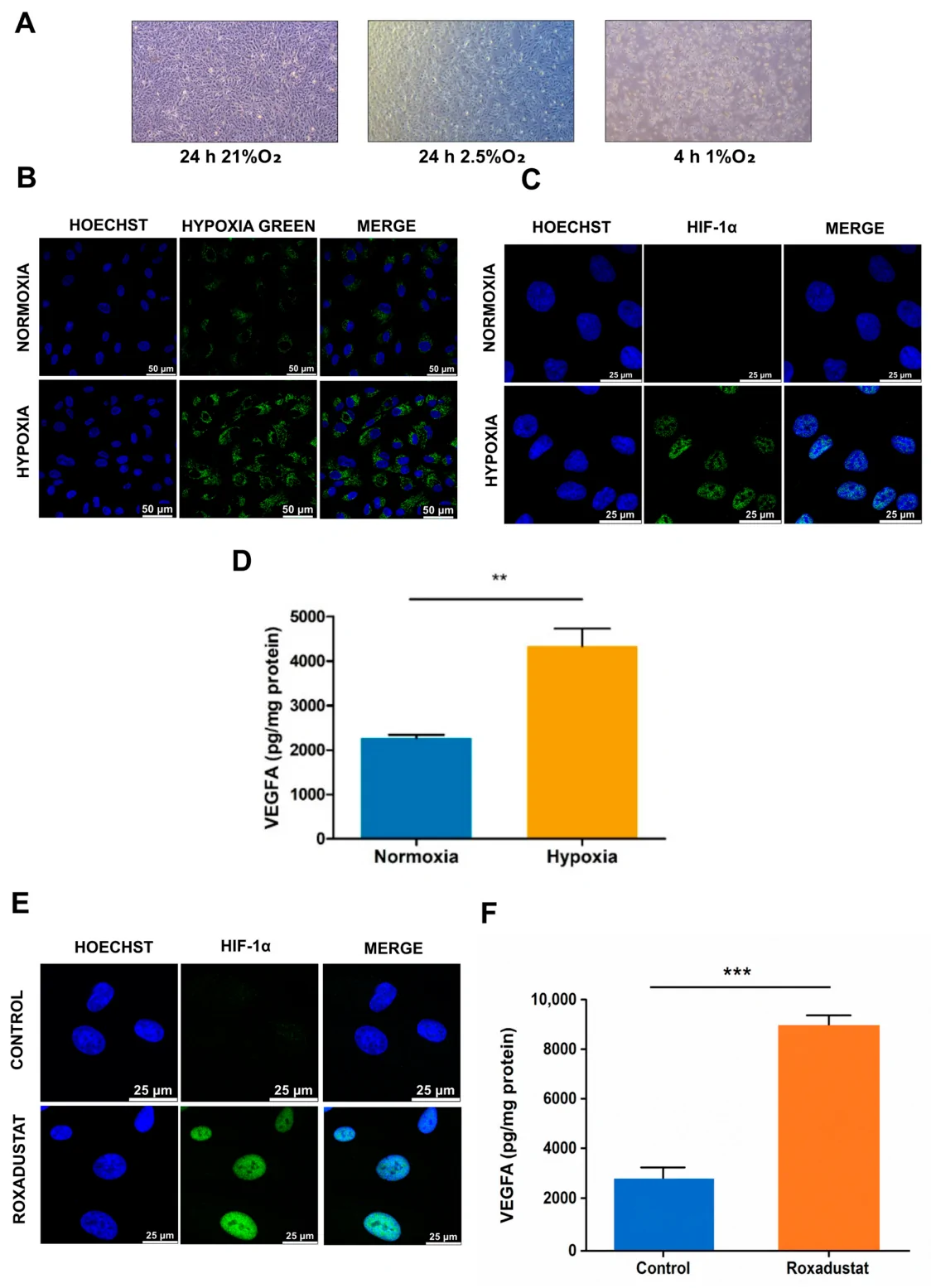
Optimization of hypoxic culture conditions for ARPE-19 cells. (**A**) ARPE-19 cells were grown at normoxia (21% O_2_) and 2.5% O_2_ for 24 h and at 1% O_2_ for 4 h. Cell morphology was assessed by brightfield microscopy. (**B**) Cells were loaded with Image-iT Green Hypoxia Reagent (green), then incubated in normoxia or hypoxia (2.5% O_2_) for 24 h. Nuclei were visualized with Hoechst (blue). (**C**) HIF-1α (green) nuclear translocation was assessed in ARPE-19 cells exposed to hypoxia (2.5% O_2_) for 24 h. Nuclei were counterstained with Hoechst (blue). (**D**) VEGFA protein levels in cell culture supernatants were measured by ELISA after 24 h in normoxia (21% O_2_) and hypoxia (2.5% O_2_). (**E**) HIF-1α (green) nuclear translocation was assessed in ARPE-19 cells exposed to Roxadustat (10 μM) for 24 h. Nuclei were counterstained with Hoechst (blue). (**F**) VEGFA protein levels in cell culture supernatants were measured by ELISA in response to Roxadustat (10 μM for 24). Data are represented as mean value ± SEM from three independent experiments. A value of *p* < 0.05 was considered statistically significant; ** *p* < 0.01, *** *p* < 0.001.

To establish a robust in vitro hypoxia model while preserving cell viability, ARPE-19 cells were cultured under 1% or 2.5% O_2_. Exposure to 1% O_2_ induced marked cytotoxicity and disrupted cell morphology within 4 h, as assessed by brightfield microscopy (Figure 1A). In contrast, culture under 2.5% O_2_ for 24 h preserved the structural integrity of the ARPE-19 monolayer (Figure 1A). Therefore, 2.5% O_2_ was selected as the hypoxic condition for all subsequent assays. To confirm that the optimized hypoxic conditions (2.5% O_2_) induced intracellular hypoxia, ARPE-19 cells were stained with Image-iT Hypoxia Green reagent. Cells maintained under normoxic conditions (21% O_2_) exhibited minimal fluorescence, whereas cells exposed to 2.5% O_2_ for 24 h showed a strong green fluorescence signal, confirming successful induction of intracellular hypoxia (Figure 1B).

To further validate the hypoxia model, we assessed the stabilization and nuclear translocation of HIF-1α, a hallmark of the cellular hypoxic response. ARPE-19 cells exposed to 2.5% O_2_ for 24 h exhibited increased HIF-1α fluorescence intensity, with prominent nuclear accumulation (Figure 1C). To assess the functional output of the hypoxic response, we quantified the secretion of vascular endothelial growth factor A (VEGFA), a well-established HIF-1α target, by enzyme-linked immunosorbent assay (ELISA). Hypoxia significantly increased VEGFA secretion into the culture supernatant compared with normoxic controls (Figure 1D). Therefore, 2.5% O_2_ was selected as the hypoxic condition for all subsequent assays. To validate pharmacological hypoxia model, we analyzed the stabilization and nuclear translocation of HIF-1α and VEGFA secretion in response to Roxadustat. ARPE-19 cells exposed to Roxadustat (10 μM) for 24 h exhibited increased HIF-1α fluorescence intensity, with prominent nuclear accumulation (Figure 1E) and significantly increased VEGFA secretion (Figure 1F). Therefore, 10 μM of Roxadustat was applied to induce pharmacological hypoxia in the subsequent experiments.

### 2.2. Comparative Transcriptomic Analysis of Physiological Hypoxia and Pharmacological HIF Activation

**Figure 2:**
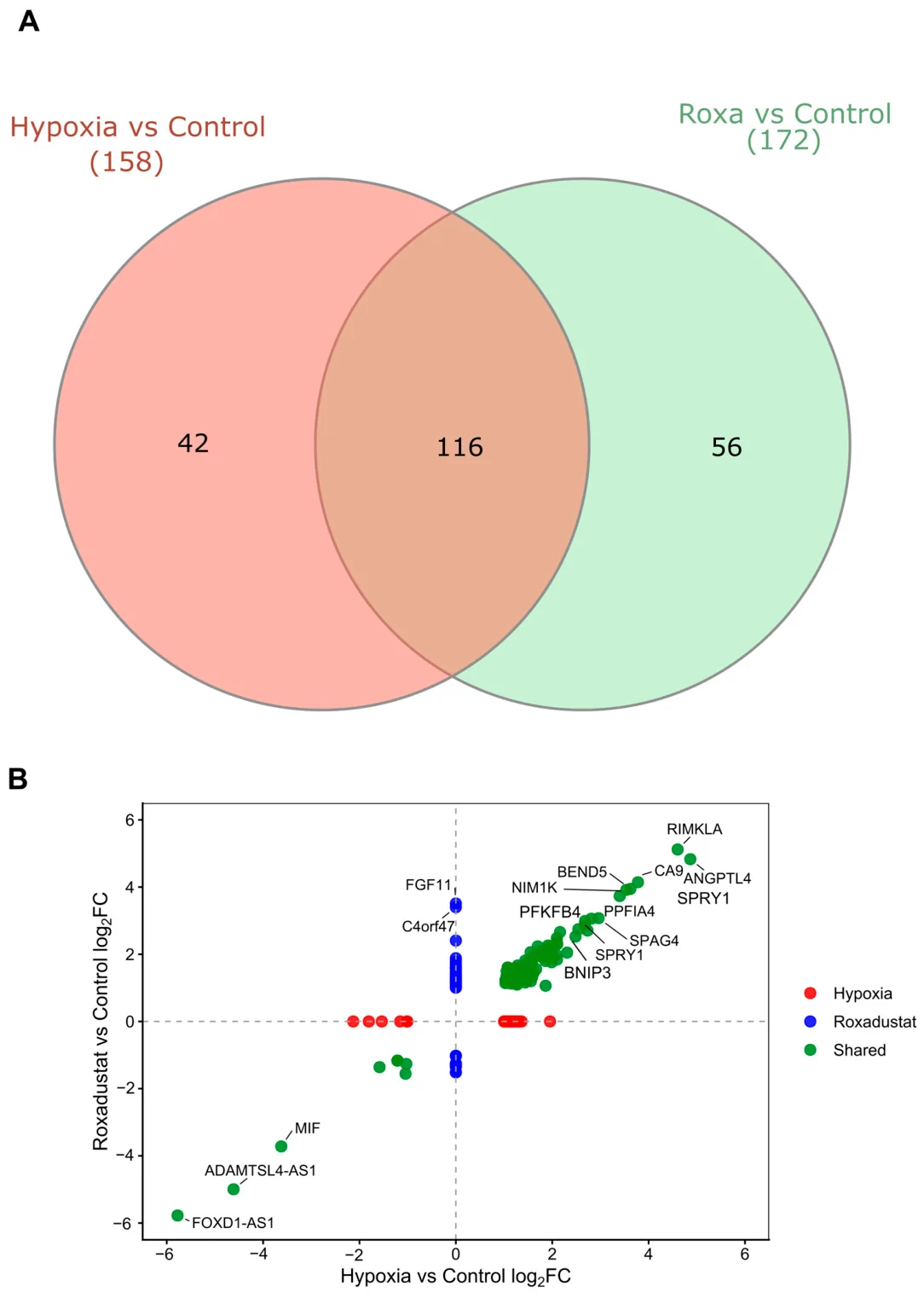
Comparative transcriptomic analysis of physiological hypoxia and pharmacological HIF activation. (**A**) Venn diagram illustrating the overlap of differentially expressed genes (DEGs) identified in hypoxia (2.5% O_2_) vs. control and HIF-PHI Roxadustat vs. control analyses. (**B**) Scatter plot comparing log_2_ fold changes (log_2_FC) between hypoxia (2.5% O_2_) vs. control (x-axis) and HIF-PHI Roxadustat vs. control (y-axis). Each point represents a gene. Shared DEGs and treatment-specific DEGs are highlighted according to Venn classification.

To distinguish transcriptional responses shared between physiological hypoxia and pharmacological HIF activation, we performed RNA-seq on ARPE-19 cells exposed to 2.5% O_2_ or treated with Roxadustat (10 μM) for 24 h. Using an adjusted *p*-value < 0.05 and log_2_FoldChange (log_2_FC) > 1 as significance thresholds, differential expression analysis identified 158 differentially expressed genes (DEGs) in hypoxia (143 upregulated and 15 downregulated) and 172 DEGs (156 upregulated and 16 downregulated) following Roxadustat treatment relative to normoxic controls. Venn analysis revealed 116 genes that were commonly regulated under both conditions, whereas 42 and 56 genes were uniquely altered by physiological hypoxia and Roxadustat treatment, respectively (Figure 2A).

To further assess the similarity between the transcriptional responses induced by physiological hypoxia and pharmacological HIF stabilization with Roxadustat, we compared the log_2_ fold changes of differentially expressed genes between the two conditions (Figure 2B). The scatter plot revealed a high degree of concordance between the two transcriptional responses, with the majority of shared DEGs showing changes in the same direction under both conditions. Most shared genes were located in the upper-right quadrant, indicating concordant upregulation, while a smaller group was located in the lower-left quadrant, indicating concordant downregulation. In contrast, only a limited number of genes were differentially expressed exclusively under physiological hypoxia or pharmacological HIF stabilization (Figure 2B). These findings indicate that pharmacological HIF stabilization largely reproduces the transcriptional response induced by hypoxia, while also revealing a small subset of condition-specific responses.

### 2.3. Functional Characterization of Shared Hypoxia-Responsive Genes

To identify the biological processes and molecular pathways represented by the genes commonly regulated by physiological hypoxia and Roxadustat treatment, we performed protein–protein interaction (PPI) network analysis and functional enrichment analysis using the STRING database. The shared gene set was analyzed for Gene Ontology (GO), Kyoto Encyclopedia of Genes and Genomes (KEGG), and Reactome pathway enrichment to identify biological processes associated with the conserved hypoxic response.

**Figure 3:**
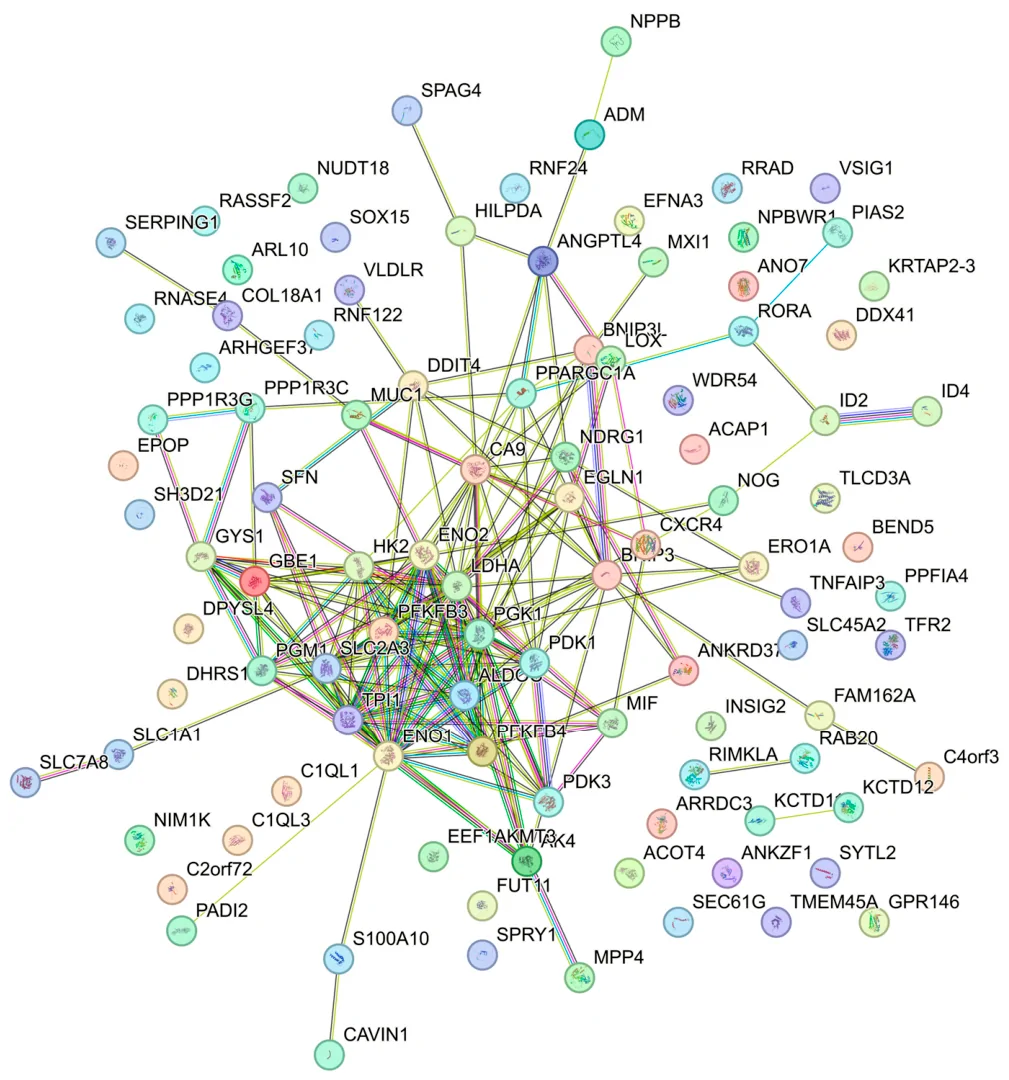
Protein–protein interaction network analysis followed by Markov Cluster Algorithm (MCL) clustering.

The STRING-based PPI network analysis demonstrated significant functional connectivity among the genes commonly regulated by hypoxia and Roxadustat treatment, indicating coordinated regulation of the shared transcriptional response. The network comprised 100 nodes and 186 edges, with interaction enrichment significantly higher than expected by chance (expected number of edges: 32) (Supplementary Figure S1). To further characterize the functional organization of the shared hypoxia- and Roxadustat-responsive genes, we performed STRING-based PPI network analysis followed by Markov Cluster Algorithm (MCL) clustering (Figure 3).

PPI analysis using MCL clustering revealed a highly interconnected core module enriched for glycolytic and hypoxia-responsive proteins, including hexokinase 2 *(HK2)*, lactate dehydrogenase A *(LDHA)*, enolase 1 *(ENO1)*, phosphoglycerate kinase 1 *(PGK1)*, pyruvate dehydrogenase kinase 1 *(PDK1)*, pyruvate dehydrogenase kinase 3 *(PDK3)*, and DNA damage inducible transcript 4 *(DDIT4)* (Figure 3). This cluster represents a coordinated metabolic program consistent with HIF-1α–mediated glycolytic reprogramming under hypoxic stress. In addition to the central metabolic module, secondary clusters included proteins involved in stress and transcriptional regulation, such as macrophage migration inhibitory factor (glycosylation-inhibiting factor) *(MIF)* and inhibitor of DNA binding *(ID)* family members, suggesting activation of inflammatory and cell-state regulatory pathways. In contrast to the densely interconnected core of upregulated hypoxia-responsive proteins, the downregulated genes Solute carrier family 45 member 2 *(SLC45A2)*, Solute carrier family 7 member 8 *(SLC7A8)*, and PPARG coactivator 1α (*PPARGC1A)* occupied peripheral positions within the network and displayed relatively few protein–protein interactions. The resulting network was dominated by a highly connected metabolic module, with the largest cluster enriched for Adenosine diphosphate (ADP) metabolism, suggesting coordinated regulation of cellular energy homeostasis. Additional functional clusters were associated with glycogen biosynthesis, amino acid transport, vascular remodeling, and hypoxia-responsive signaling (Figure 3). Notably, a distinct cluster containing canonical hypoxia-associated genes, including carbonic anhydrase 9 *(CA9)*, egl-9 family hypoxia-inducible factor 1 *(EGLN1)*, and lysyl oxidase *(LOX)*, was identified, supporting activation of the HIF-dependent O_2_ response pathway (Figure 3). Together, these findings indicate that the shared transcriptional signature induced by hypoxia and Roxadustat treatment is organized around conserved metabolic adaptation and O_2_-response programs.

**Figure 4:**
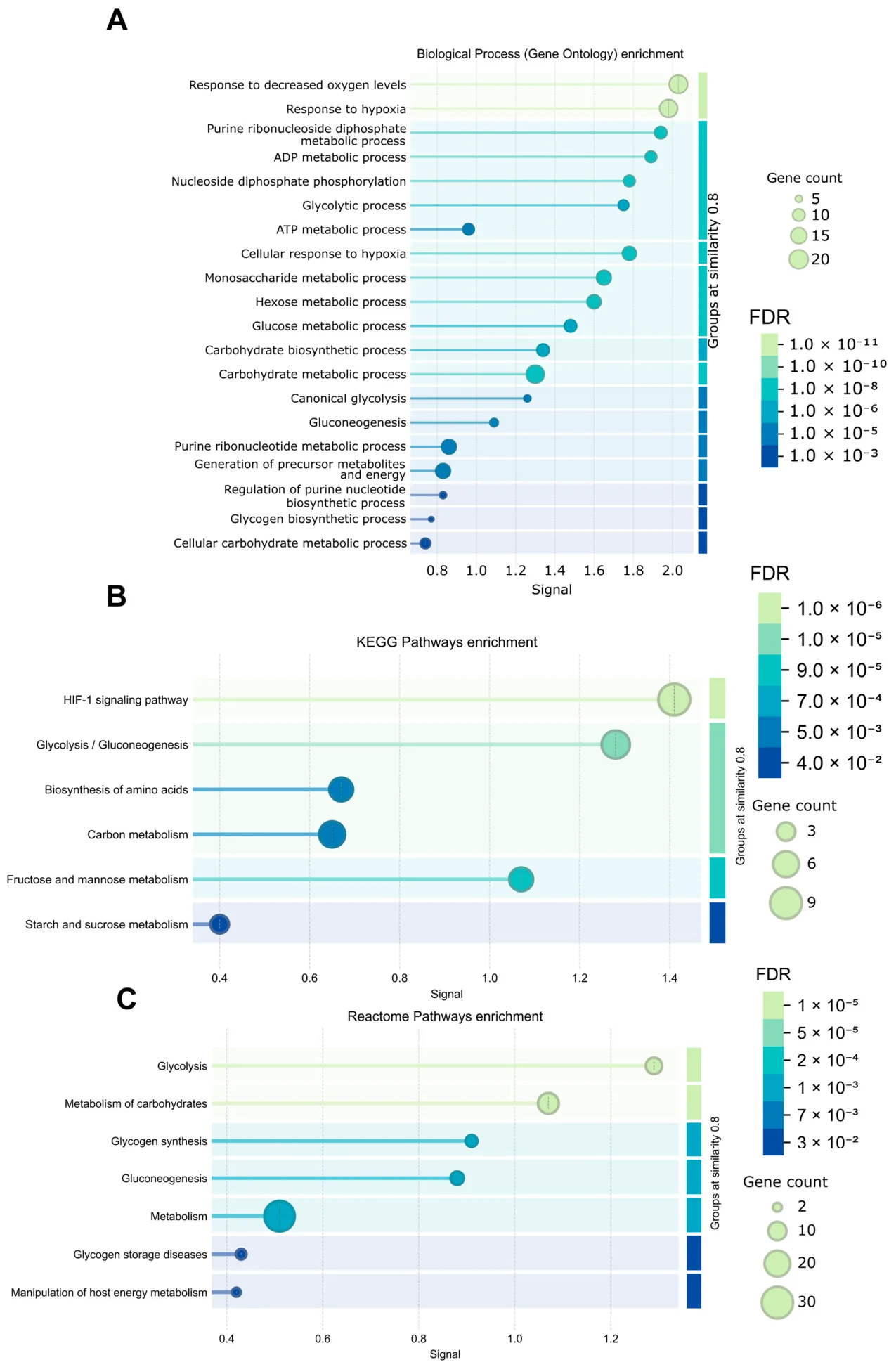
Functional characterization of shared hypoxia-responsive genes. (**A**) Gene Ontology Biological Process enrichment showing significantly overrepresented functional categories. (**B**) Kyoto Encyclopedia of Genes and Genomes (KEGG) pathway enrichment identifying canonical signaling and metabolic pathways. (**C**) Reactome pathway enrichment providing high-resolution mapping of molecular pathways. Enrichment was based on adjusted significance thresholds (FDR < 0.05).

GO Biological Process analysis revealed that shared DEGs were significantly enriched in biological processes related to key cellular regulatory functions, including response to decreased O_2_ level, response to hypoxia, the purine ribonucleoside diphosphate metabolic process, the ADP metabolic process, and carbohydrate metabolism (Figure 4A).

KEGG pathway analysis further supported this observation, identifying enrichment of canonical signaling and metabolic pathways involved in HIF-1 signaling pathways, glycolysis/gluconeogenesis, biosynthesis of amino acids, carbon metabolism, fructose and mannose metabolism, and starch and sucrose metabolism (Figure 4B). The consistency of enriched pathways across both treatments indicates robust activation of shared molecular mechanisms. Reactome pathway analysis provided higher-resolution functional annotation and confirmed enrichment of pathways associated with glycolysis, metabolism of carbohydrates, glycogen synthesis, gluconeogenesis, metabolism, glycogen storage diseases, and manipulation of host-energy metabolism (Figure 4C).

### 2.4. GO Enrichment Reveals Metabolic and Hypoxia-Related Biological Processes

**Figure 5:**
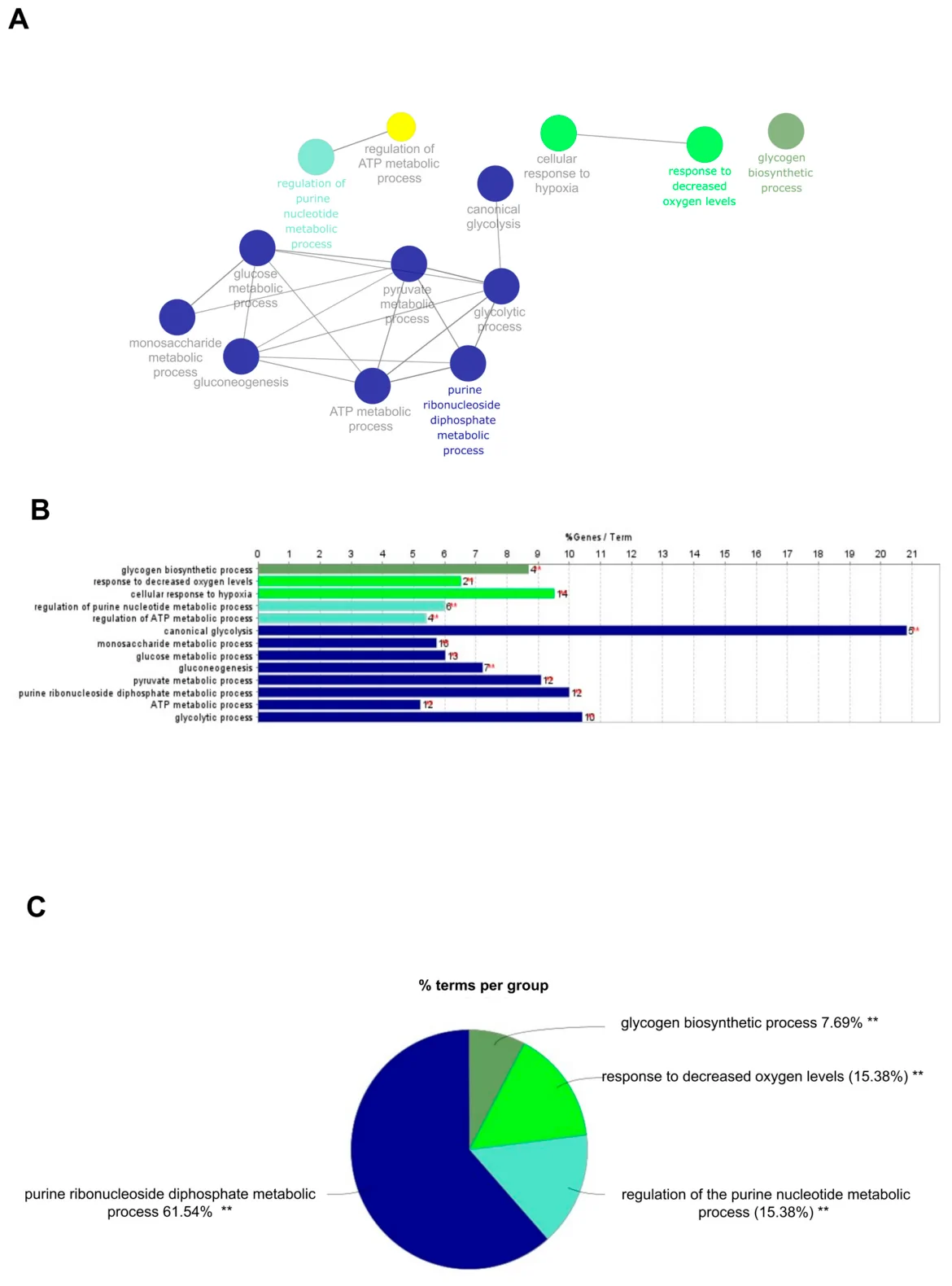
ClueGO network analysis of shared DEGs. (**A**) GO term network showing nodes (GO terms) and edges representing functional similarity based on kappa score. Functionally related terms are grouped into clusters and color-coded. (**B**) Functional group pie chart illustrating the proportion of GO terms assigned to each major biological cluster. (**C**) GO functional groups and their associated biological processes. ** *p* < 0.01.

To further investigate the functional relationships among enriched biological processes, GO term network analysis was performed on the shared DEGs using ClueGO. This approach was used to complement standard enrichment analysis by grouping related GO Biological Process terms into functionally coherent modules. ClueGO analysis initially identified 61 significantly enriched GO terms (Supplementary Figure S2). Following application of more stringent filtering criteria, 13 representative terms remained (Figure 5A). These terms were primarily associated with the purine ribonucleoside diphosphate metabolic process, regulation of purine nucleotide metabolism, response to decreased O_2_ levels, and the glycogen biosynthetic process (Figure 5A). The relative contribution of each functional group is shown in a pie chart (Figure 5B).

Among the enriched biological processes, canonical glycolysis exhibited the highest enrichment, with approximately 21% of the input genes (5 genes) associated with this pathway, as well as an additional significantly enriched response to decreased O_2_ levels (21 genes), cellular response to hypoxia (14 genes), and the glycolytic process (10 genes) (Figure 5B). The distribution of enriched GO terms further demonstrated that the purine ribonucleoside diphosphate metabolic process constituted the largest functional category, accounting for 61.54% of the enriched terms (Figure 5C). This was followed by response to decreased O_2_ levels (15.38%) and regulation of the purine nucleotide metabolic process (15.38%), whereas the glycogen biosynthetic process represented the smallest category (7.69%) (Figure 5C). Collectively, these findings indicate that the differentially expressed genes are predominantly involved in energy metabolism, nucleotide metabolism, carbohydrate metabolism, and cellular adaptation to hypoxia.

### 2.5. Differential Gene Expression Analysis Between Roxadustat and Hypoxia

**Figure 6:**
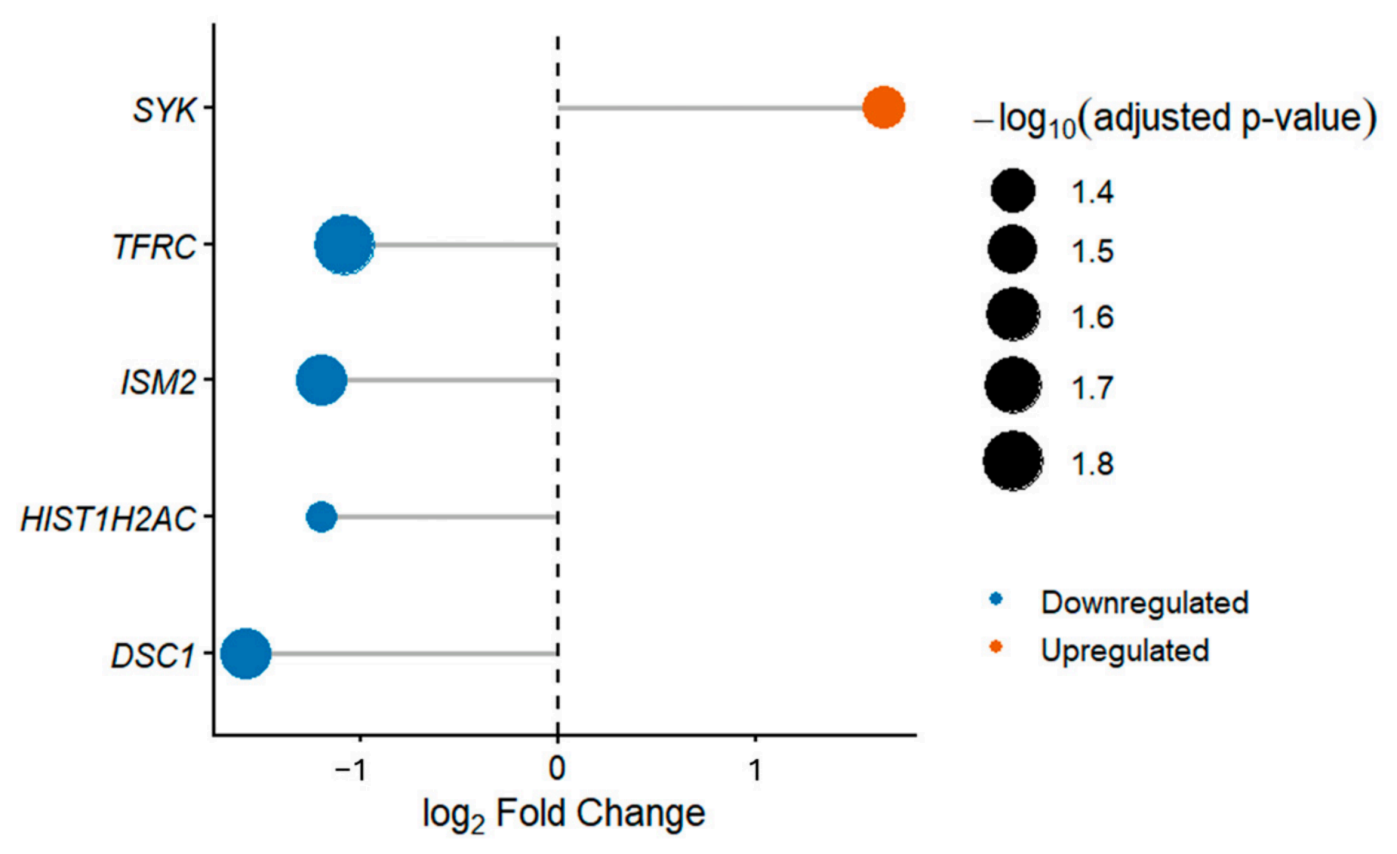
Lollipop analysis of differentially expressed genes between Roxadustat and hypoxia.

Differential expression analysis comparing Roxadustat and hypoxia identified five significantly changing DEGs. Specifically, spleen-associated tyrosine kinase *(SYK)* was significantly upregulated in Roxadustat, whereas transferrin receptor *(TFRC)*, isthmin 2 *(ISM2)*, histone cluster 1 H2A family member c *(HIST1H2AC)*, and desmocollin 1 *(DSC1)* were significantly downregulated relative to hypoxia. The lollipop plot summarizes the direction and magnitude of expression changes for the five significant DEGs (Figure 6).

Overall, only a small number of genes exhibited significant differential expression between Roxadustat and hypoxia, suggesting that the transcriptomic differences between the two conditions were modest under the applied statistical criteria. To further investigate the potential biological functions of the five genes that were differentially expressed between physiological hypoxia and pharmacological HIF stabilization, functional enrichment analysis was performed using ClueGO v2.5.10. No significantly enriched Gene Ontology (GO) Biological Process terms or pathways were identified for this gene set. Consistent with these findings, analysis using the STRING online database also did not identify significant functional enrichment or pathway associations.

### 2.6. Effects of Inhibitors on VEGFA Secretion and Tube Formation Potential of Hypoxic ARPE-19 Cells

To determine whether LY294002, GN44028, and Rapamycin could mitigate the cellular hypoxic response, ARPE-19 cells were subjected to LY294002 (50 μM), GN44028 (10 μM), and rapamycin (100 nM) for 1 h in normoxia, then transferred to hypoxia or treated with Roxadustat (10 μM) in the presence or absence of the inhibitors for 24 h.

**Figure 7:**
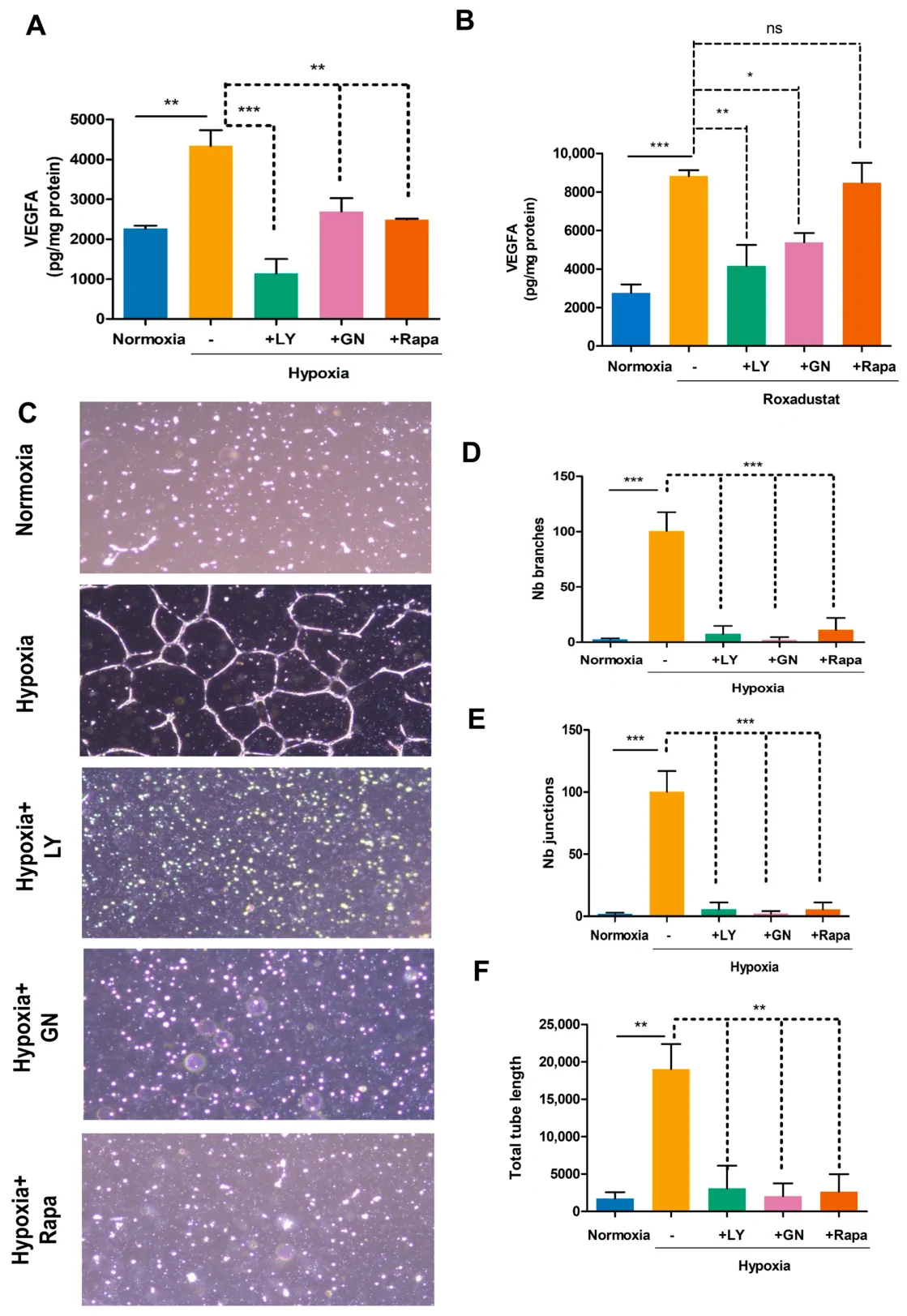
Effects of inhibitors on VEGFA secretion and tube formation potential of hypoxic ARPE-19 cells. (**A**) VEGFA protein levels in hypoxic cell culture supernatants measured by ELISA after 24 h. (**B**) VEGFA protein levels in Roxadustat-treated cell culture supernatants measured by ELISA after 24 h. (**C**) Endothelial cell tube formation assay using ARPE-19-conditioned media ube formation was visualized using a Leica DMi1 microscope. (**D**) Number (Nb) of branches, (**E**) Nb of junctions, and (**F**) total tube length analyzed by Fiji software v. 1.54p with Angiogenesis Analyzer plugin. (* *p* < 0.05, ** *p* < 0.01, *** *p* < 0.001).

ELISA results showed a robust decrease in VEGFA protein expression in the culture supernatant of inhibitor-treated cells compared to the hypoxia control group (Figure 7A). In contrast to this, in the Roxadustat-treated cells, LY294002 and GN44028 but not rapamycin significantly decreased VEGFA production (Figure 7B).

To determine whether inhibition of hypoxia-induced VEGFA production translated into a functional angiogenic outcome, human umbilical vein endothelial cell (HUVEC) tube formation assays were performed using conditioned media from ARPE-19 cells exposed to hypoxia in the presence or absence of LY294002, GN44028, and Rapamycin. ARPE-19 cells cultured under normoxic conditions served as the control group. We showed that consistent with the induction of pro-angiogenic pathways by hypoxia, HUVECs exposed to the conditioned media of hypoxic ARPE-19 cells exhibited a robust increase in capillary-like tube formation on the Geltrex matrix compared to those treated with normoxic control-conditioned media (Figure 7C). Notably, the addition of all three tested inhibitors significantly decreased the tube formation of HUVECs stimulated by the hypoxic ARPE-19-conditioned media (Figure 7C).

Each inhibitor successfully disrupted the capillary network, as evidenced by a marked reduction in key tube formation parameters, including number of branches (Figure 7D), number of junctions (Figure 7E), and total tube length (Figure 7F) on the gel matrix. These data demonstrate that LY294002, GN44028, and rapamycin effectively blunts the pro-angiogenic signaling cascade triggered by hypoxic ARPE-19 cells.

## 3. Discussion

Our findings indicate that pharmacological HIF stabilization with Roxadustat in ARPE-19 cells induces several molecular responses that are also observed under hypoxic conditions. However, the differential response of VEGFA to pharmacological HIF stabilization suggests that HIF activation alone may not fully reproduce the regulation of VEGFA that occurs during physiological hypoxia. These findings highlight the possibility that additional oxygen-sensitive mechanisms contribute to the cellular response to hypoxia.

Selection of an appropriate hypoxic condition is critical, as excessive O_2_ deprivation may compromise cell viability and confound downstream functional analyses. Consistent with this, exposure to 1% O_2_ rapidly induced cytotoxicity and compromised morphology of ARPE-19 cells, whereas 2.5% O_2_ preserved cellular architecture while eliciting a robust hypoxic response. The coordinated increase in intracellular hypoxia and nuclear localization of HIF-1α demonstrated that 2.5% O_2_ was sufficient to activate the canonical hypoxia signaling pathway without causing overt cellular damage. Collectively, these findings indicate that this model recapitulates the key molecular features of hypoxia while providing a stable and physiologically relevant platform for investigating hypoxia-dependent mechanisms in RPE cells.

Comparative analysis of the transcriptional responses to physiological hypoxia and HIF-PHI Roxadustat revealed a high degree of concordance between the two conditions. Although Venn analysis identified treatment-specific DEGs, comparison of log_2_FC values demonstrated that most genes were regulated in a similar direction and to a comparable extent, indicating that pharmacological HIF stabilization largely recapitulates the core transcriptional response to hypoxia. It should be noted that the apparent treatment-specific genes identified in the scatter plot are defined by statistical significance thresholds rather than the complete absence of transcriptional changes in the alternate condition. Nevertheless, the distribution of these genes suggests that a limited subset may be differentially regulated by physiological O_2_ deprivation or pharmacological HIF stabilization. Together, these findings indicate that the transcriptional responses to hypoxia and Roxadustat are largely shared, while also supporting the possibility of subtle condition-specific regulatory differences.

The MCL-based PPI network demonstrates that hypoxic stress in ARPE-19 cells induces a modular reorganization of interaction networks. The dominant module reflects a canonical glycolytic switch, characterized by coordinated upregulation of enzymes involved in glucose metabolism and pyruvate handling, consistent with HIF-1-driven metabolic adaptation.

Peripheral nodes such as SLC45A2, SLC7A8, and PPARGC1A were less densely connected in the PPI network, suggesting that HIF activation is accompanied not only by activation of a central glycolytic module but also by suppression of genes associated with epithelial identity and metabolic specialization. In particular, SLC45A2, a melanosomal transporter essential for pigmentation, was downregulated; SLC45A2 knockout leads to increased acidification of early melanosomes, thereby affecting tyrosinase activity and ultimately limiting melanin synthesis [19], which mitigates damage to the retina and internal nerves from ultraviolet light [20]. Similarly, downregulation of SLC7A8, an L-type amino acid transporter expressed in RPE cells [21], suggests reduced amino acid transport capacity and altered nutrient handling under metabolic stress. Notably, SLC7A8 has also been reported to protect human RPE cells against ornithine-induced cytotoxicity [22]. Together, these findings indicate that reduced SLC7A8 expression may compromise both amino acid homeostasis and cellular stress resistance in RPE cells. This may further exacerbate metabolic vulnerability under hypoxic conditions, contributing to impaired cellular function and survival. PGC-1α reduces oxidative stress, a key factor in age-related macular degeneration (AMD) pathogenesis associated with cellular senescence, through the upregulation of antioxidant enzymes and DNA damage response pathways [23]. In addition, PGC-1α is an important regulator of VEGFA, a major therapeutic target in neovascular (wet) AMD, the most severe form of the disease [23]. Decreased PPARGC1A expression in response to hypoxia and HIF-PHI treatment may indicate suppression of mitochondrial biogenesis and oxidative metabolic programs. Glycolytic shift represented resulting in increased glucose utilization in the RPE is implicated in photoreceptor degeneration [24,25]. Several genes upregulated under both physiological hypoxia and pharmacological HIF stabilization, including LOX [26] and PDK1 [27,28], have been implicated as key contributors to the progression of retinopathies. Together, these changes support a model in which hypoxia and HIF-PHI induces a coordinated transition from a differentiated, metabolically flexible RPE phenotype toward a glycolysis-dominant state with reduced cellular specialization.

Importantly, only five genes (*DSC1*, *HIST1H2AC*, *ISM2*, *SYK*, and TFRC) were significantly differentially expressed in the direct comparison between Roxadustat and physiological hypoxia, further supporting the conclusion that the two treatments elicit highly similar transcriptional programs. This finding suggests that the treatment-specific DEGs identified in the Venn analysis are, at least in part, attributable to significance thresholds applied in the individual comparisons rather than reflecting fundamentally distinct biological responses. Nevertheless, these genes may represent specific molecular pathways that distinguish the cellular response to the two treatments. DSC1, a cadherin involved in calcium-dependent cell–cell adhesion, is a component of desmosomes [29] may contribute to the maintenance of epithelial integrity in retinal pigment epithelial cells. Alterations in DSC1 expression under hypoxic conditions may reflect remodeling of intercellular junctions and reduced epithelial stability, consistent with stress-induced disruption of RPE monolayer.

*HIST1H2AC* encodes a replication-dependent histone H2A protein involved in nucleosome assembly and chromatin organization. Although its specific role in RPEs has not been established, differential expression of HIST1H2AC may reflect chromatin remodeling associated with hypoxia-induced transcriptional reprogramming [30]. Such epigenetic changes are increasingly recognized as important regulators of RPE responses to oxidative and metabolic stress [31].

ISM2, a protein which expression in humans is almost specific to the placenta, with no well-defined role in RPEs [32]. Although its biological function in the RPE remains unclear, it has both angiogenic and anti-angiogenic activity [32]. Further studies are required to determine its functional significance.

Epithelial–mesenchymal transition (EMT) of retinal pigment epithelial (RPE) cells is a characteristic feature of the progression of AMD. Activation of spleen-associated tyrosine kinase (SYK) and Src proto-oncogene, non-receptor tyrosine kinase (SRC), promotes the migration and invasion of RPE cells in response to cigarette smoke extract, thereby facilitating EMT [33]. Furthermore, pharmacological inhibition of SYK and SRC effectively suppressed VEGFA production in cigarette smoke extract-treated ARPE-19 cells, while also reducing the expression of mesenchymal markers and cell migratory activity [33]. Therefore, increased SYK expression in response to pharmacological hypoxia may promote RPE dysfunction by facilitating EMT and stimulating VEGFA production, thereby contributing to pathological angiogenesis and disease progression in AMD.

Downregulation of TFRC, which encodes the transferrin receptor responsible for cellular iron uptake, suggests altered iron homeostasis in hypoxic RPE cells in pharmacological hypoxia compared to physiological hypoxia. Given that iron-mediated oxidative stress may contribute to retinal degeneration in AMD [34], reduced TFRC expression may represent an adaptive response to limit iron-dependent oxidative stress by decreasing intracellular iron accumulation in pharmalogical hypoxia.

VEGFA is a canonical HIF target gene and a major mediator of angiogenesis, and plays a pivotal role in the pathogenesis of DR [35], AMD [36], and ROP [37]. Previously, we have found that HIF-PHIs stabilize HIF under normoxic conditions in RPEs [38], and that assessment of VEGFA expression provides an important indicator of HIF pathway activation and its potential impact on RPE function.

The differential effects of the inhibitors on VEGFA production may reflect distinct mechanisms of HIF pathway activation. While hypoxia stabilizes HIF, HIF-PHIs directly inhibit prolyl hydroxylase enzymes, resulting in sustained HIF stabilization independent of O_2_ tension [39]. In our previous study, we demonstrated that LY294002, the hypoxia inhibitor BAY 87-2243, and rapamycin suppress hemorrhage-induced metabolic reprogramming and the angiogenic potential of RPE cells [14]. In the present study, LY294002 and GN44028 significantly reduced VEGFA production under both hypoxic and HIF-PHI-treated conditions, suggesting that their inhibitory effects occur downstream of, or independently from, the mechanism of HIF stabilization. In contrast, rapamycin attenuated VEGFA production only under hypoxic conditions and failed to suppress HIF-PHI-induced VEGFA expression, indicating that its inhibitory activity may depend on hypoxia-specific signaling events that are not engaged during pharmacological HIF activation. Collectively, these findings suggest that hemorrhage- and hypoxia-induced VEGFA production may share common signaling pathways, whereas HIF-PHI-induced VEGFA expression is regulated, at least in part, through distinct molecular mechanisms. Further studies are required to elucidate the pathways underlying these differential responses.

To determine whether the reduction in hypoxia-induced VEGFA production translated into a functional anti-angiogenic effect, conditioned media from inhibitor-treated hypoxic ARPE-19 cells were assessed using a HUVEC tube formation assay. As expected, conditioned media from hypoxic ARPE-19 cells markedly enhanced endothelial tube formation compared with normoxic controls, confirming that hypoxic RPE cells secrete factors that promote angiogenesis. Importantly, conditioned media from ARPE-19 cells treated with LY294002, GN44028, or rapamycin significantly impaired capillary-like network formation, as demonstrated by reductions in the number of branches, junctions, and total tube length. These findings indicate that all three inhibitors effectively attenuate the pro-angiogenic activity of hypoxic RPE cells. The observed inhibition of endothelial tube formation is consistent with the reduced VEGFA production detected in inhibitor-treated hypoxic ARPE-19 cells, suggesting that suppression of VEGFA contributes to the diminished angiogenic response. However, because RPE cells secrete a broad range of angiogenic and inflammatory mediators in response to hypoxia, it is likely that the inhibitors also influence additional factors that regulate endothelial cell behavior. Therefore, the reduced tube-forming capacity observed in HUVECs probably reflects the combined effects of altered secretion of multiple hypoxia-responsive mediators rather than changes in VEGFA alone. Collectively, these findings demonstrate that inhibition of PI3K signaling by LY294002, HIF signaling by GN44028, and mTOR signaling by rapamycin effectively suppresses the pro-angiogenic phenotype induced by hypoxia in RPE cells. By reducing the ability of hypoxic RPE cells to stimulate endothelial network formation, these inhibitors may represent potential therapeutic strategies for limiting pathological angiogenesis associated with retinal diseases characterized by chronic hypoxia, including neovascular age-related macular degeneration.

Physiological hypoxia and pharmacological HIF stabilization elicit highly similar, yet distinct, responses in ARPE-19 cells. Both conditions activated a conserved HIF-dependent transcriptional program characterized by the induction of glycolytic and hypoxia-responsive genes and increased VEGFA production, while direct comparison identified only a limited number of DEGs. Nevertheless, the differential effects of pharmacological inhibitors indicate that physiological hypoxia engages additional O_2_-sensitive signaling pathways beyond HIF stabilization alone.

The present study has several limitations. First, the experiments were performed using ARPE-19 cells, which provide a convenient and widely used in vitro model of RPE biology but do not fully reproduce the phenotype and functional complexity of primary or native human RPE. Second, the hypoxic conditions were investigated in a simplified cell culture system. Although 2.5% O_2_ induced a robust hypoxic response while preserving cellular morphology, this experimental model cannot fully recapitulate the complex interactions between RPE cells, photoreceptors, retinal vasculature, and other cell types present in the intact retina. Third, pharmacological HIF stabilization with Roxadustat does not reproduce all molecular consequences of physiological hypoxia. Furthermore, the direct comparison between physiological hypoxia and pharmacological HIF stabilization identified only five significantly differentially expressed genes. Although these genes may provide clues to condition-specific mechanisms, their functional relevance in RPE cells remains incompletely established, and additional experimental validation is required.

Collectively, these findings indicate that HIF-PHIs recapitulate the core HIF-dependent hypoxic response but do not fully reproduce the complexity of physiological hypoxia, providing important insights into RPE adaptation to O_2_ deprivation and the biological effects of pharmacological HIF activation. Future studies should aim to delineate these HIF-independent O_2_-sensitive pathways and determine their functional contribution to metabolic reprogramming and angiogenic signaling in RPE cells.

## 4. Materials and Methods

### 4.1. Reagents

Unless otherwise stated, we obtained all reagents from Sigma-Aldrich (St. Louis, MO, USA). Roxadustat was purchased from Cayman Chemical (Ann Arbor, MI, USA) and dissolved in dimethyl sulfoxide (DMSO). LY294002, GN44028, and Rapamycin were obtained from MedChemExpress (Bergkällavägen, Sollentuna, Sweden) and dissolved in DMSO.

### 4.2. Cell Culture and Hypoxic Treatment

The human retinal pigment epithelial cell line (ARPE-19; Cat. No. CRL-2302) was purchased from the American Type Culture Collection (ATCC; Manassas, VA, USA). Cells were maintained in Dulbecco’s Modified Eagle Medium/Nutrient Mixture F-12 (DMEM/F-12; Capricorn Scientific, Ebsdorfergrund-Dreihausen, Germany) supplemented with 5% fetal bovine serum (FBS, Capricorn Scientific, Ebsdorfergrund-Dreihausen, Germany), 100 U/mL penicillin, 100 μg/mL streptomycin, and amphotericin B. Experimental procedures were restricted to cells between passages 6 and 8. To induce hypoxic stress, cells were cultured under hypoxic conditions (2.5% O_2_, 5% CO_2_, and 92.5% N_2_) within a Hypoxic Glove Box (Coy Laboratory Products, Grass Lake, MI, USA) for durations ranging from 3 to 24 h, depending on the specific experimental design. In some experiments, cells were incubated under hypoxic conditions (1% O_2_, 5% CO_2_, and 94% N_2_) for 4 h. For inhibitor studies, cells were pre-treated with either LY294002 (50 μM), Rapamycin (100 nM), or GN44028 (10 μM) under normoxic conditions for 1 h prior to 24 h exposure to hypoxia. To induce pharmacological hypoxia, cells were exposed to Roxadustat (10 μM) for 24 h. For inhibitor studies, cells were pre-treated with either LY294002 (50 μM), Rapamycin (100 nM), or GN44028 (10 μM) for 1 h prior to 24 h exposure to Roxadustat (10 μM). Inhibitors were present during the 24 h Roxadustat treatments.

### 4.3. Image-iT Green Hypoxia Staining

To evaluate cellular hypoxia, ARPE-19 cells were seeded onto 35 mm glass-bottom dishes (Ibidi, Gräfelfing, Germany) and incubated overnight at 37 °C in a humidified CO_2_ incubator until they achieved approximately 90% confluence. The following day, the culture medium was aspirated and replaced with fresh FluoroBrite DMEM (Thermo Fisher Scientific, Waltham, MA, USA) supplemented with 5% FBS, 100 U/mL penicillin, 100 μg/mL streptomycin, and amphotericin B, containing 5 µM Image-iT™ Green Hypoxia Reagent (Thermo Fisher Scientific, Waltham, MA, USA). Following a 30 min incubation at 37 °C, the cells were washed with phosphate-buffered saline (PBS, pH 7.4) to remove excess reagent. The cells were then maintained in complete FluoroBrite DMEM and exposed to either normoxic (21% O_2_ or hypoxic (2.5% O_2_) conditions for 3 h. Live-cell imaging and fluorescence analysis were subsequently performed using a Leica SP8 confocal microscope equipped with Leica Application Software X (v1.4.7.28982; Leica, Mannheim, Germany).

### 4.4. Immunofluorescent Staining

Immunofluorescent staining was performed as described earlier [38]. Briefly, cells were grown on coverslips and exposed to hypoxia (2.5% O_2_ for 24 h) or Roxadustat (10 μM for 24 h) as described above. Cells were washed with PBS pH 7.4 twice and fixed with 4% paraformaldehyde solution in PBS for 15 min at 37 °C. Coverslips were then washed three times with PBS pH 7.4, then blocked in Blocking Buffer (5% normal goat serum/0.3% Triton X-100 in PBS) for 60 min. Primary antibodies against HIF-1α (dilution 1:400) (Cell Signaling Technology, Danvers, MA, USA) were diluted in an antibody dilution buffer (1% BSA/0.3% Triton X-100 in PBS) and incubated overnight at 4 °C. Samples were then incubated then with goat anti-rabbit IgG conjugated to Alexa Fluor 488 (Thermo Fisher Scientific, Waltham, MA, USA) (Thermo Fisher Scientific, Waltham, MA, USA) at a dilution of 1:500 for 60 min. Nuclei were visualized with Hoechst (Thermo Fisher Scientific, Waltham, MA, USA). Samples were investigated with Lightning super-resolution microscopy using Leica Application Software X (v1.4.7.28982; Leica, Mannheim, Germany).

### 4.5. VEGFA ELISA

Supernatants from ARPE-19 cells exposed to hypoxia (2.5% O_2_) and HIF-PHI Roxadustat (10 μM) were collected 24 h after the treatments and measured using Human VEGF DUOSet ELISA (R&D Systems, Abingdon, UK). VEGFA expressions were normalized to protein content determined using the bicinchoninic acid assay (Pierce BCA Protein Assay Kit, Thermo Fisher Scientific, Waltham, MA, USA).

### 4.6. RNA-Seq Method

Total RNA was extracted 24 h post-treatment using TriReagent (Zymo Research, Irvine, CA, USA). Global transcriptome profiling was conducted through mRNA sequencing using the Illumina platform as described earlier [38]. RNA was isolated from three independent biological replicates for each experimental condition, including normoxia, hypoxia, and pharmacological hypoxia (*n* = 3 per condition). RNA concentration and purity were assessed by measuring the absorbance ratios at 260/280 nm and 260/230 nm. Only samples meeting the appropriate quality criteria were used for subsequent RNA-seq analysis. Total RNA sample quality was checked on 2100 BioAnalyzer using Eukaryotic Total RNA Nano Kit (Agilent, Santa Clara, CA, USA) according to the manufacturer’s protocol. Samples with RNA integrity number (RIN) value > 8 were accepted for library preparation process. RNA-Seq libraries were prepared from total RNA using Ultra II RNA Sample Prep kit (New England BioLabs, Ispwich, MA, USA) according to the manufacturer’s protocol. Briefly, poly-A mRNAs were captured by oligo-dT conjugated magnetic beads, then the mRNAs were eluted and fragmented at 94-Celsius degree. First-strand cDNA was generated by random priming reverse transcription and after second-strand synthesis step double-stranded cDNA was generated. After repairing ends, A-tailing and adapter ligation steps were performed, adapter ligated fragments were amplified in enrichment PCR, and finally sequencing libraries were generated. RNA-Seq libraries were prepared from total RNA using a Ultra II RNA Sample Prep kit (New England BioLabs, Ipswich, MA, USA) according to the manufacturer’s protocol. Sequencing runs were executed on the Illumina NextSeq 2000 instrument (Illumina, San Diego, CA, USA). Single-read 100 bp long sequencing was performed; the base calling accuracy Q30 was >93%. The average read number was 25.1 million reads per samples; the read numbers varied between 21.5 and 28.9 million. Raw sequencing reads were aligned to the human reference genome version GRCh38; the alignment percentage was >95%. Aligned sequencing data have been deposited into the NCBI SRA database under accession no. PRJNA1496033.

### 4.7. RNA-Seq Data Analysis

RNA-seq data analysis was performed as described previously [14]. Briefly, raw sequencing reads in FASTQ format were aligned to the human reference genome (GRCh38) using HISAT2, generating BAM files. Downstream analysis was performed using StrandNGS software v3.4 (Strand Life Sciences Pvt. Ltd., Bangalore, India). Data were normalized using the DESeq algorithm, and differential gene expression between conditions was assessed using a moderated *t*-test. A *p*-value < 0.05 was considered statistically significant.

### 4.8. Venn Analysis

Venn analysis was performed with Interactivenn online tool (https://www.interactivenn.net/ accessed on 24 June 2026) [40].

### 4.9. Comparative Transcriptomic Analysis

To assess the relationship between transcriptional responses induced by physiological hypoxia and pharmacological hypoxia, log_2_FC values of genes detected in both comparisons (hypoxia vs. normoxia control and Roxadustat vs. normoxia control) were compared. A scatter plot was generated in RStudio v2026.06.0 (Posit team (2026). RStudio: Integrated Development Environment for R. PositSoftware, PBC, Boston, MA. URL http://www.posit.co/ accessed on 18 August 2026 software using ggplot2 v4.0.3 [41], plotting log_2_FC in hypoxia vs. control (x-axis) against log_2_FC in Roxadustat vs. control (y-axis). Shared and treatment-specific DEGs were highlighted based on Venn classification. Lollipop plots were generated in RStudio and ggplot2 package as described above. Each gene was represented by a point connected to the zero baseline, illustrating both the magnitude and direction of differential expression.

### 4.10. Functional Enrichment Analysis of Shared Differentially Expressed Genes

Shared differentially expressed genes (DEGs), identified in both hypoxia vs. control and HIF-PHI Roxadustat vs. control comparisons, were subjected to functional enrichment analysis. GO Biological Process, KEGG pathway, and Reactome pathway analyses were performed using STRING online database (v12.0 https://string-db.org/ accessed on 20 August 2026). Enrichment significance was assessed using a false discovery rate (FDR) threshold of <0.05. Results were visualized to identify overrepresented biological processes and signaling pathways associated with the shared transcriptional response.

### 4.11. Pathway Analyses

Cytoscape v3.10.4 [42], along with the ClueGO v2.5.10 [43] plugin, was utilized to identify significantly enriched Gene Ontology (GO) terms. A two-sided hypergeometric test with Benjamini–Hochberg correction was conducted to compare the list of differentially expressed genes against the GO Biological Process database.

### 4.12. Endothelial Cell Tube Formation Assay

The formation of endothelial cell tube networks was analyzed using an Angiogenesis Starter Kit (GIBCO, Thermo Fisher Scientific, Waltham, MA, USA) as described earlier [14]. To produce conditioned culture media, ARPE-19 cells were cultured under hypoxic condition (2.5% O_2_) in DMEM/F12 supplemented with 2% FBS for 24 h. Then, supernatants were concentrated 25-fold using Amicon Ultra Centrifugal Filter, 10 kDa (Merck, Darmstadt, Germany) according to the manufacturer’s guide. Endothelial tube formation assay (ln Vitro Angiogenesis) was performed as recommended by the Angiogenesis Starter Kit. Human umbilical vein endothelial cells (HUVECs) were cultured in Medium 200 supplemented with Large VesseI Endothelial Supplement (LVES). Several 35 mm glass-bottom dishes (Ibidi, Gräfelfing, Germany) were coated with 155 μL of Geltrex LDEV-Free Reduced Growth Factor Basement Membrane Matrix according to the manufacturer’s guide. HUVECs were trypsinized and resuspended in Medium 200 containing 2% FBS without LVES. Then, 75,000 cells (in a maximal final volume of 110 μL) in Medium supplemented with 2% FBS without LVES were plated in 1 mL of 25-fold concentrated conditioned supernatants from ARPE-19 cells and incubated for 16 h. Tube formation was observed using the Leica DMi1 microscope. Tube formation analysis was performed with Fiji software v1.54p [44] using the Angiogenesis Analyzer plugin [45].

### 4.13. Statistical Analysis

Statistical analysis was carried out by one-way ANOVA test followed by Bonferroni correction or *t*-test using GraphPad Prism 5.0 software (GraphPad Software, Boston, MA, USA). A value of *p* < 0.05 was considered statistically significant (* *p* < 0.05, ** *p* < 0.01, *** *p* < 0.001). Data are represented as mean value ± SEM from three independent experiments. For RNA-Seq data analysis, a moderated *t*-test was used to determine differentially expressed genes between conditions, where *p* < 0.05 was considered a significant difference.

## Data Availability

The original contributions presented in this study are included in the article/Supplementary Materials. Further inquiries can be directed to the corresponding author.

## Supplementary Materials

The following supporting information can be downloaded at: https://www.mdpi.com/article/10.3390/ph19091355/s1, Figure S1: STRING-based PPI network analysis of the shared genes. Figure S2: STRING-based PPI network analysis of the shared genes.

## Abbreviations

The following abbreviations are used in this manuscript:

ADP	Adenosine diphosphate
AMD	Age-related macular degeneration
ATCC	American Type Culture Collection
CA9	Carbonic anhydrase 9
CKD	Chronic kidney disease
DDIT4	DNA damage inducible transcript 4
DEG	Differentially expressed gene
DMEM/F12	Dulbecco’s Modified Eagle Medium/Nutrient Mixture F-12
DMSO	Dimethyl sulfoxide
DR	Diabetic retinopathy
DSC1	Desmocollin 1
EGLN1	Egl-9 family hypoxia-inducible factor 1
ELISA	Enzyme-Linked Immunosorbent Assay
ENO1	Enolase 1
FBS	Fetal bovine serum
GO	Gene Ontology
HIF	Hypoxia-inducible factor
HIF-1α	Hypoxia-inducible factor-1α
HIF-PHI	Hypoxia-inducible factor–prolyl hydroxylase domain inhibitors
HIST1H2AC	Histone cluster 1 H2A family member c
HK2	Hexokinase 2
HUVEC	Human umbilical vein endothelial cell
ID	Inhibitor of DNA binding
ISM2	Isthmin 2
KEGG	Kyoto Encyclopedia of Genes and Genomes
LDHA	Lactate dehydrogenase A
LOX	Lysyl oxidase
LVES	Large vesseI endothelial supplement
MCL	Markov Cluster Algorithm
MIF	Macrophage migration inhibitory factor (glycosylation-inhibiting factor)
PBS	Phosphate-buffered saline
PDK1	Pyruvate dehydrogenase kinase 1
PDK3	Pyruvate dehydrogenase kinase 3
PGK1	Phosphoglycerate kinase 1
PPARGC1A	PPARG coactivator 1 alpha
PPI	Protein–protein interaction
RPE	Retinal pigment epithelium
SLC45A2	Solute carrier family 45 member 2
SLC7A8	Solute carrier family 7 member 8
SYK	Spleen-associated tyrosine kinase
TFRC	Transferrin receptor
VEGF	Vascular endothelial growth factor
