# Supplementary material for: Physiological Hypoxia and Pharmacological HIF Stabilization Induce Distinct but Overlapping Responses in Retinal Pigment Epithelial Cells
Source: Pharmaceuticals (Basel). 2026 Aug 27;19(9):1355. doi: 10.3390/ph19091355 (PMC13610669; doi:10.3390/ph19091355)
Supplement: Supplementary file 1 [file pharmaceuticals-19-01355-s001.zip › pharmaceuticals-4473094-supplementary.pdf]

Article

Physiological Hypoxia and Pharmacological HIF Stabilization Induce Distinct but Overlapping Responses in Retinal Pigment Epithelial Cells

Tamás Gáll [1], Dávid Pethő [1,2], Szilárd Póliska [3], József Balla [1,2]* and György Balla [2,4]

[1] Department of Internal Medicine, Division of Nephrology, Faculty of Medicine, University of Debrecen, H-4032 Debrecen, Hungary

[2] HUN-REN–UD Vascular Biology and Myocardium Pathophysiology Research Group, Hungarian Academy of Sciences, University of Debrecen, H-4032 Debrecen, Hungary

[3] Genomic Medicine and Bioinformatic Core Facility, Department of Biochemistry and Molecular Biology, Faculty of Medicine, University of Debrecen, H-4032 Debrecen, Hungary

[4] Department of Pediatrics, Faculty of Medicine, University of Debrecen, H-4032 Debrecen, Hungary

* Author to whom correspondence should be addressed.
balla@belklinika.com
Tel.: +36 52 255 600 / 55004
Department of Internal Medicine, Division of Nephrology, Faculty of Medicine, University of Debrecen, H-4032 Debrecen, Hungary

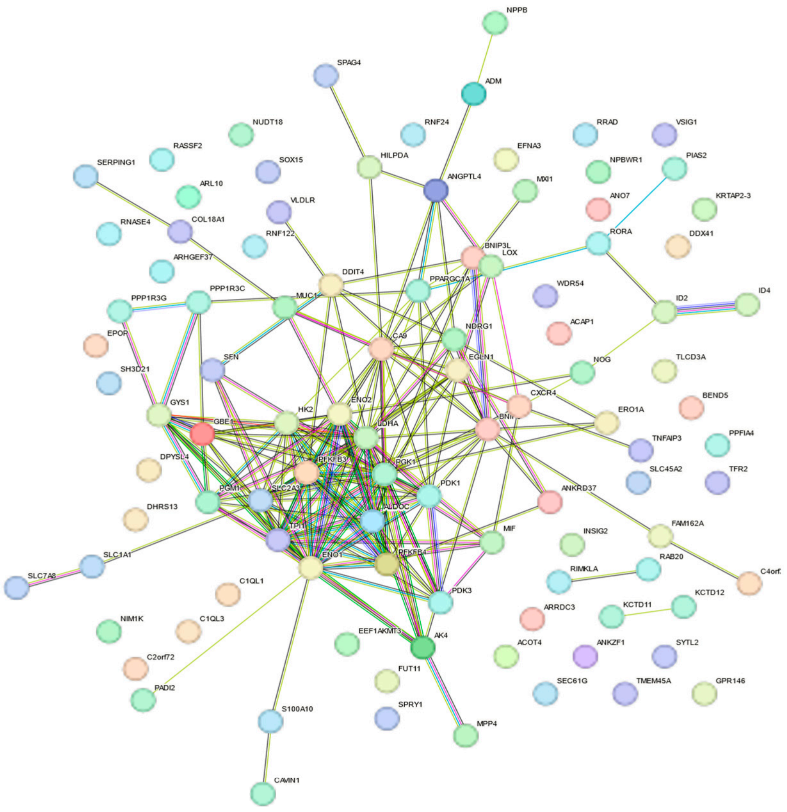

Supplementary Figure S1. STRING-based PPI network analysis of the shared genes.

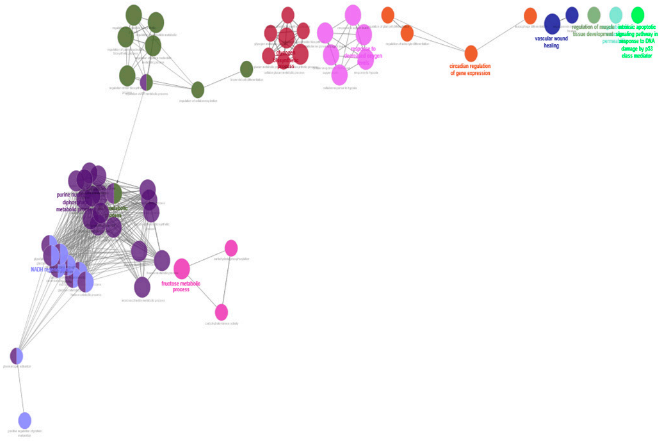

Supplementary Figure S2. STRING-based PPI network analysis of the shared genes.
